# Recombinant Antibody Production Using a Dual-Promoter Single Plasmid System

**DOI:** 10.3390/antib10020018

**Published:** 2021-05-13

**Authors:** Stefania C. Carrara, David Fiebig, Jan P. Bogen, Julius Grzeschik, Björn Hock, Harald Kolmar

**Affiliations:** 1 Institute for Organic Chemistry and Biochemistry, Technische Universität Darmstadt, Alarich-Weiss-Str. 4, D-64287 Darmstadt, Germany; stefania.carrara@ferring.com (S.C.C.); fiebig@biochemie-tud.de (D.F.); bogen@biochemie-tud.de (J.P.B.); 2 Ferring Darmstadt Laboratories, Alarich-Weiss-Str. 4, D-64287 Darmstadt, Germany; julius.grzeschik@ferring.com; 3 Ferring International Center S.A, Chemin de la Vergognausaz 50, CH-1162 Saint Prex, Switzerland; bjorn.hock@ferring.com

**Keywords:** monoclonal antibodies, promoters, bidirectional, antibody production, upstream processing

## Abstract

Monoclonal antibodies (mAbs) have demonstrated tremendous effects on the treatment of various disease indications and remain the fastest growing class of therapeutics. Production of recombinant antibodies is performed using mammalian expression systems to facilitate native antibody folding and post-translational modifications. Generally, mAb expression systems utilize co-transfection of heavy chain (*hc*) and light chain (*lc*) genes encoded on separate plasmids. In this study, we examine the production of two FDA-approved antibodies using a bidirectional (BiDi) vector encoding both *hc* and *lc* with mirrored promoter and enhancer elements on a single plasmid, by analysing the individual *hc* and *lc* mRNA expression levels and subsequent quantification of fully-folded IgGs on the protein level. From the assessment of different promoter combinations, we have developed a generic expression vector comprised of mirrored enhanced CMV (eCMV) promoters showing comparable mAb yields to a two-plasmid reference. This study paves the way to facilitate small-scale mAb production by transient cell transfection with a single vector in a cost- and time-efficient manner.

## 1. Introduction

With the growing interest in monoclonal antibodies (mAbs) for therapeutic applications, advances in antibody production have improved drastically over the last decades. Due to the more complex structure of antibodies, their production requires host cells capable of natively folding and modifying the mAb. Modifications include post-translational glycosylation, which is, among other functional properties, critical to reduce their immunogenicity [1]. For this purpose, mammalian cells fulfil the requirements as appropriate hosts for antibody production [2,3]. Advances in transfection protocols and cell engineering have boosted the use of suspension cell lines with the ability to grow at high densities, and increased production yields [4,5]. Further within the drug discovery and development process, stable cell lines are generated for the most promising candidate(s), while transient transfection is performed at earlier stages to yield research quantities of mAbs, sufficient for characterization of lead candidates. The accessibility of commercially available transfection reagents with high efficiencies and the use of disposable materials makes transient expression an efficient and cost-effective strategy during early drug discovery [6]. Human Embryonic Kidney 293 (HEK293) and Chinese Hamster Ovary (CHO) cells are commonly used for transient antibody expression, due to their high expression yields and human-like glycosylation patterns [1,7,8]. 

The basis of successful antibody production is the correct folding of individual chains, followed by their accurate assembly, resulting in functional heterotetrameric glycoproteins. Misfolded or partially folded antibodies are degraded by the host cell’s intrinsic quality control system, resulting in low production yields. Furthermore, antibodies with undesired folding are not able to effectively engage their target antigen or to mediate effector functions, have unfavourable pharmacokinetics, and tend to aggregate. Besides these biological limitations, purification of antibody products contaminated with aggregated or misfolded mAbs is a major hindrance in the downstream processing of therapeutic molecules and is currently the topic of numerous studies [9,10,11,12]. 

Antibody folding starts upon co-translational translocation into the endoplasmic reticulum (ER) [13]. Following the homodimerization of the two heavy chains (HC), the light chains (LC) are associated and covalently linked via disulphide bonds [14]. The glycosylation at Asn297 is linked to the CH2 backbone in a co-translational manner [15]. During translation, chaperones are involved to ensure the correct folding of the individual domains, as well as the final assembly of the tetrameric mAb [16].

At present, the largest part of transient production of mAbs is carried out using a two-plasmid system, also known as co-transfection, for the expression of *lc* and *hc*, with each gene driven by its own promoter and transcribed separately [17,18]. These are carried out, for the most part, with an equimolar ratio of heavy chain and light chain genes. Nonetheless, contradictory results have been reported using Expi293-F cells. While some publications report that an equimolar gene ratio results in the highest yield of fully assembled IgGs [8], others have described optimal expression with a 1:2 ratio of heavy and light chain genes, respectively [19].

A large drawback of the two-plasmid system is the moderate control over the relative expression of *lc* and *hc*, with fluctuating cell-to-cell transfection efficiencies [17]. On the other hand, a bidirectional (BiDi) vector with a dual-promoter can ensure the introduction of both *hc* and *lc* genes into each cell in equal amounts. However, for some applications it could be beneficial to have a stronger expression of one chain over the other. By choosing two suitable promoters controlling the transcription of *hc* and *lc*, different ratios can be achieved [20]. While diverse approaches have been developed throughout the years to advance and facilitate recombinant antibody production, the design of expression vectors plays a large role for optimization of expression yields. Recently, Bayat and colleagues (2018) compared the use of three different vector design strategies for the expression of IgG1 antibodies in CHO cells, namely using the conventional two-vector approach with *hc* and *lc* encoded separately, a bicistronic vector based on internal ribosome entry sites (IRES), and a dual-promoter single vector approach. All vectors were under the control of a human cytomegalovirus (CMV) promoter. Expression analysis revealed that the dual-promoter vector system resulted in the highest mAb yield [21].

Andersen and co-workers have previously shown the ability of the CMV enhancer to control two core CMV promoters simultaneously, resulting in efficient antibody expression [22]. With this basis, we sought to investigate different promoters in a bidirectional format to facilitate transient transfection, avoiding co-transfections. We also sought to simplify *hc* and *lc* gene cloning by establishing a one-step Golden Gate cloning procedure that relies on the simultaneous plasmid incorporation of *hc* and *lc* genes together with the bidirectional promoter sequence. By analysing *hc* and *lc* gene expression, as well as through subsequent quantification of fully-folded IgGs, promoter and enhancer element combinations were compared. Here, we show the use of a dual-promoter, single plasmid approach using divergent promoters for the transient expression of two FDA-approved antibodies, Durvalumab and Avelumab, using both Expi293-F and ExpiCHO-S cell systems. This work lays the foundation to facilitate small-scale mAb production in drug discovery programs in a more efficient manner.

## 2. Materials and Methods

### 2.1. Plasmids & Cloning of Constructs

**Figure 1:**
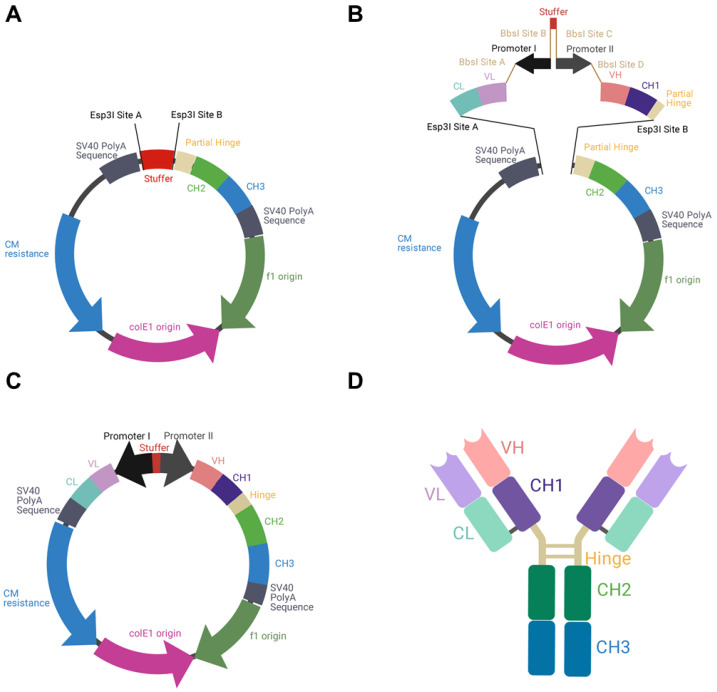
Schematic illustration of BiDi promoter system for antibody production. (**A**) The MD vector was designed to exhibit a 200-bp stuffer, flanked by *Esp*3I restriction sites (*Esp*3I sites A and B), adjacent to a SV40 polyA signal sequence and the regions encoding for hinge-CH2-CH3, terminated by a SV40 polyA signal sequence. (**B**) VL-CL and VH-CH1 amplicons can be inserted into MD by Golden Gate cloning utilizing *Esp*3I restriction sites (*Esp*3I sites A and B). The BiDi promoter can be chosen individually and is flanked by *Bbs*I sites (*Bbs*I sites A–D), compatible with the VL and VH sequences. (**C**) Golden Gate assembly results in a fully functional and re-circularized vector, with the light chain under the control of promoter I and the heavy chain under the control of promoter II. (**D**) Schematic representation of the resulting heterotetrameric IgG1 antibody using the same colour code as for the genetic elements.

To allow the individual exchange of different variable domains, the utilization of κ and λ isotypes, as well as the usage of different BiDi promoter combinations, the backbone of the mammalian destination (MD) vector was built in a cassette-like manner. For vector amplification in *E. coli*, a chloramphenicol resistance was utilized, adjacent to the colE1 and the f1 origins. A stuffer sequence, flanked by *Esp*3I restriction sites, was downstream of an inverse-orientated SV40 polyA sequence that was intended to be a terminator signal for the light chain cassette. Upstream of the stuffer sequence, a partial hinge followed by the CH2 and CH3 domains of a human IgG1 were encoded. Again, a SV40 polyA signal sequence served as a terminator signal (Figure 1A). The plasmid was de novo designed in silico and ordered at GeneArt (Regensburg, Germany).

The selected promoter sequences were either ordered as gene strings at Twist Bioscience (EF-1α, minCMV-enh-CMV (GenBank: MK764037) or PCR-amplified from the pTT5 CMV promoter cassette between bases 42–1185 (hereinafter referred to as eCMV) [23]. To allow for the correct orientation of the promoter sequences, individual primers were used to introduce the respective *Bbs*I Golden Gate cloning (GGC) signature overhangs. Genes for VH-CH1 and VL-CL of Durvalumab (Imfinzi, κ light chain) and Avelumab (Bavencio, λ light chain) were also ordered as gene strings, already bearing suitable signature sequences for *Esp*3I and *Bbs*I as well as their respective leader sequences. The 200-bp stuffer used between individual promoters consists of the non-functional 3′ coding region of the amp resistance gene for beta-lactamase, followed by ~40 bp of non-coding DNA. Assembly of the MD expression constructs was conducted with 75 ng destination vector and equimolar amounts of the respective fragments, 20 U *Bbs*I-HF, 10 U *Esp*3I, and 200 U T4-DNA ligase (NEB, Frankfurt, Germany) for 30 cycles (1 min; 16 °C; 37 °C). For the reference constructs, VH and VL genes were amplified incorporating *Sap*I restriction sites and then inserted into a pTT5-derived vector utilizing CH1-CH2-CH3 or κ/λ entry vectors using GGC as described before [24,25]. PCR reactions were performed utilizing Q5 polymerase (NEB) according to the manufacturer’s protocol and purified using the Wizard SV Gel and PCR Clean-up System (Promega, Walldorf, Germany). All primers can be found in Table S1. The DNA sequence for the 2xeCMV BiDi construct can be found in Sequence S1 in the Supplementary Information.

*E. coli* XL1-blue were transformed utilizing the Golden Gate reaction mixtures and cultivated on chloramphenicol or ampicillin DYT agar plates for MD or pTT5 constructs, respectively. Resulting colonies were sequenced at MicroSynth SeqLab (Göttingen, Germany), and positive clones were utilized to inoculate 50 mL overnight cultures. Plasmid DNA for transient transfection was isolated using the PureYield Plasmid Midiprep System (Promega, Walldorf, Germany).

### 2.2. Cell Lines

Expi293-F and ExpiCHO-S cells were obtained from Thermo Fisher Scientific. Cells were incubated at 37 °C, 8% CO_2_, 110 rpm, and sub-passaged every 3–4 days in their respective expression media, as described in the manufacturer’s protocol (Thermo Fisher Scientific, Schwerte, Germany). Cell count and viability were measured using an automated cell counter (Bio-Rad TC-20) based on trypan blue staining. Cell densities were maintained between 0.3–4 × 10^6^ cells/mL and 0.2–6 × 10^6^ cells/mL for Expi293-F and ExpiCHO-S, respectively.

### 2.3. 24-Well Transfection

For gene expression and protein quantification, small-scale transfections using Axygen 24-well deep-well plates (Corning, New York, NY, USA) were performed. One day prior to transfection, cells were seeded into wells at a final cell density of 1.8 × 10^6^ or 3 × 10^6^ viable cells/mL in 2.5 mL expression medium for Expi293-F or ExpiCHO-S, respectively, and incubated under shaking conditions in a humified atmosphere at 37 °C, 8% CO_2_, 225 rpm. The following day, the cell density was adjusted to 3 × 10^6^ or 6 × 10^6^ viable cells/mL in 2.5 mL expression medium for Expi293-F or ExpiCHO-S, respectively. DNA:Expifectamine complexes were incubated at room temperature with either 3 µg BiDi plasmid or 2 µg heavy and 2 µg light chain plasmid for co-transfections (two-plasmid reference) for 20 or 1 min for Expi293-F or ExpiCHO-S, respectively, before adding dropwise to the cells. Feeding procedures were carried out according to manufacturer’s instructions. For gene expression analysis, cells were harvested 3 days post-transfection, while protein quantification was carried out 6 days post-transfection.

### 2.4. RNA Isolation

Three days post-transfection, Expi293-F or ExpiCHO-S cells were harvested by centrifugation and cell pellets were processed through a QIAshredder column (QIAGEN, Hilden, Germany). Total RNA extraction was carried out using RNeasy Mini Kit (QIAGEN) following the manufacturer’s instructions. RNA concentration was determined spectroscopically using a NanoDrop One (Thermo Fisher), ensuring pure RNA was isolated with a A260/280 ratio of 2.0.

### 2.5. Gene Expression Analysis by Reverse Transcription Quantitative Polymerase Chain Reaction (RT-qPCR)

Expression levels of heavy and light chain (both κ and λ) were analysed using 100 ng RNA per well in Hard-Shell^®^ 96-well PCR plates (Bio-Rad, Hercules, CA, USA) and iTaq Universal SYBR Green One-step Kit (Bio-Rad) with designed SYBR Green primers (Sigma Aldrich, Munich, Germany) using a CFX96 qPCR instrument (Bio-Rad). Relative expression levels were analysed using the integrated software from Bio-Rad (CFX Manager, Hercules, CA, USA) and normalized to housekeeping genes GAPDH and RPLP0 (IDT, Coralville, IA, USA). The primers used can be found in Table S2.

### 2.6. Protein Purification

To purify the antibodies from small-scale transfections, cells were harvested by centrifugation and cell culture supernatants were purified using Protein A HP SpinTrap columns (Cytiva, Freiburg im Breisgau, Germany) following the manufacturer’s protocol. Antibodies were eluted in 0.1 M glycine-HCl, pH 2.7. Protein concentration was determined using a NanoDrop One (Thermo Fisher) using the corresponding molecular weights and extinction coefficients.

### 2.7. Protein Quantification and Affinity Determination Using Biolayer Interferometry (BLI)

Six days post-transfection, cells were harvested by centrifugation and the cell culture supernatants were sterile-filtered (0.45 µm). BLI experiments were performed on an Octet Red96 (FortéBio, Fremont, CA, USA). Using Protein A biosensors (Sartorius, Göttingen, Germany) for quantification, mAb concentration was measured from the cell culture supernatants. An in-house produced mAb was used as a standard within the range of 3.13–400 µg/mL.

For affinity determination, anti-human Fab-CH1 2nd generation (FAB2G) biosensors (Sartorius) were used. Purified antibodies were loaded onto the tips at 10 µg/mL until a layer thickness of 1 nm was reached. Association was measured using a serial dilution of His-PD-L1-TwinStrep (produced in-house). Kinetics were determined using Savitzky-Golay filtering and a 1:1 Langmuir binding model.

## 3. Results

To produce full-length antibodies in a bidirectional manner, we first designed the respective vector in silico. This vector encoded the fragment crystallizable (Fc) region of an IgG1, which is the most common isotype found in therapeutic antibodies [26]. In order to allow the flexible use for a variety of binders, the fragment antigen binding (Fab), which can be of the κ or λ isotype, was not encoded on the plasmid. Instead, a stuffer sequence, flanked by *Esp*3I sites was incorporated (Figure 1A).

As a reference antibody to establish different promoter combinations, the Fab of Durvalumab, an FDA-approved anti-PD-L1 antibody of the κ type, was chosen [27]. PCR amplicons of VH-CH1 and VL-CL were generated introducing *Esp*3I and *Bbs*I restriction sites. By utilization of a BiDi promoter system, flanked by *Bbs*I sites, a Golden Gate reaction resulted in a re-circularized vector (Figure 1B,C). In this process, the stuffer that was included in the parental MD vector was replaced by a CL-VL-PromoterI-Stuffer-PromoterII-VH-CH1 sequence. Owing to this cloning strategy, a straightforward exchange of different Fabs and different BiDi promoters is feasible. The resulting vector exhibited the functional ORFs for the heavy and the light chains, resulting in the production of full-length antibodies (Figure 1D).

### 3.1. Cloning Promoter Combinations

Based on the findings from Andersen et al. [22] that a single enhancer adjacent to a bidirectional CMV promoter allows for efficient antibody production using a single expression plasmid, six different bidirectional promoter complexes were generated comprising not only the minCMV-CMV cassette, but also combinations of other strong promoters including their individual enhancer elements, such as the CMV with its major immediate early enhancer (MIE), the optimized CMV cassette from pTT5 (denoted as eCMV in this study), and the human translation elongation factor 1 alpha (EF-1α) promoter, that were selected based on their capability to produce fully-folded IgG molecules.

The MIE-CMV promoter, being one of the most commonly used in mammalian expression vectors, is designated as a strong driver for recombinant protein expression [28,29]. As shown before by Andersen and colleagues [22], the MIE enhancer is also capable of facilitating elevated expression levels in the divergently oriented minCMV promoter, although to a lesser extent. This was based on the presumption that the formation of the large transcription complex might be sterically hindered. As a reference, we selected a similar bidirectional promoter setup lacking the unique sequence upstream of the enhancer. Additionally, we went for a mirror-symmetric approach comprised of two individual CMV promoters, each having adjacent MIE enhancers that were separated by a 200-bp-stuffer. The eCMV cassette comprises—besides the MIE enhancer and core promoter—several additional regulatory elements that have been described to increase expression levels. These elements include the non-coding adenoviral tripartite leader sequence (TPL), the adenovirus major late promoter enhancer (MLP enh.), as well as distinct splicing sites allowing for prolonged mRNA stability [30,31]. Furthermore, we also utilized the strong human translation elongation factor 1 alpha promoter (EF-1α) that has proven to be advantageous over the CMV promoter in some cell types and in the expression of distinct proteins of interests [32,33].

**Figure 2:**
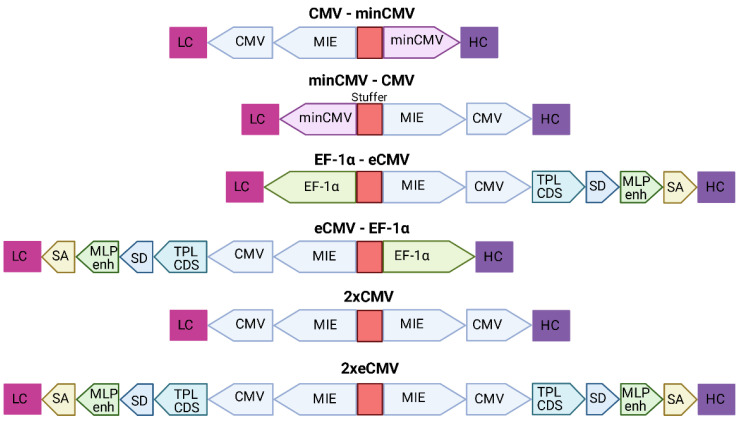
Overview of the different bidirectional combinations tested. The 200-bp stuffer sequence is marked in red for each construct. Abbreviations: minimal CMV (minCMV), cytomegalovirus promoter (CMV), enhanced CMV (eCMV), major immediate early enhancer (MIE), human translation elongation factor 1 alpha (EF-1α), adenoviral tripartite leader sequence (TPL CDS), adenovirus major late promoter enhancer (MLP enh.), splicing donor site (SD), splicing acceptor site (SA), light chain (LC), heavy chain (HC).

Based on the modular setup of our MD vector and utilization of GGC, we generated different combinations of the aforementioned promoters to analyse for highest full-length antibody expression levels and product yield of Durvalumab. A schematic overview of the BiDi combinations is depicted in Figure 2.

### 3.2. Gene Expression Analysis in Mammalian Cells

**Figure 3:**
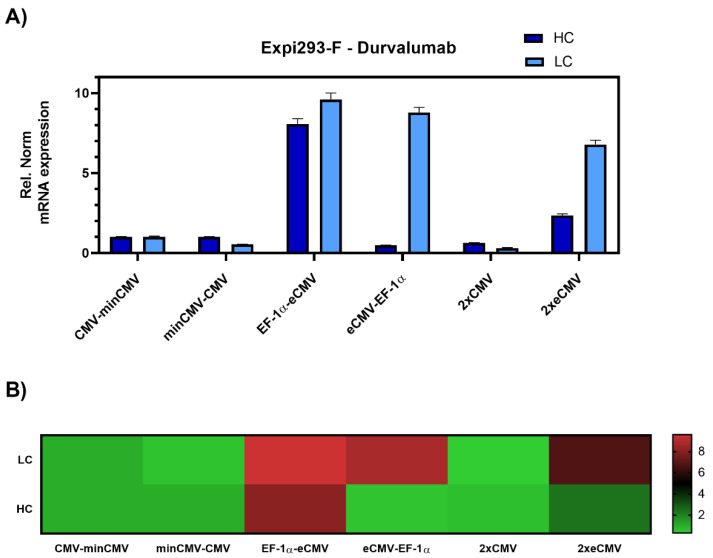
Gene expression analysis of heavy and light chain genes after transient transfection of Durvalumab in Expi293-F cells. (**A**) Bar chart representing heavy (dark blue) and light (light blue) chain mRNA expression in the different constructs. Values are relative to the CMV-minCMV construct and normalised to housekeeping genes GAPDH and RPLP0. Error bars represent the standard error of the mean of technical triplicates. (**B**) Heat map representation of gene expression analysis. The relative normalised gene expression for light and heavy chain mRNA is shown on the right.

Transient transfections of all six promoter combinations were performed in 24-well plates using Expi293-F cells, an established cell line for transient antibody production. Three days post-transfection, cells were harvested, and RNA was isolated. Relative gene expression of *hc* and *lc* mRNA levels was measured by RT-qPCR (Figure 3).

The data was set relative to the CMV-minCMV construct and normalised to housekeeping genes. Looking at relative mRNA levels, both variants with minCMV and CMV, independent of the promoter orientation, did not yield high mRNA expression for either *lc* or *hc*. Remarkably, the combinations with the EF-1α promoter and the enhanced CMV cassette (eCMV) showed significant differences in expression levels depending on their orientation. Steering *lc* expression with the EF-1α promoter (EF-1α-eCMV) resulted in a 9.5-fold upregulation in *lc* and 8-fold upregulation in *hc* mRNA levels. Conversely, having the more potent eCMV in the light chain direction and EF-1α in the heavy chain direction (eCMV-EF-1α) led to a 9-fold *lc* upregulation, and low relative *hc* expression levels. Based on these results, mirrored constructs containing both promoter and enhancer cassettes in both directions were tested, one with the traditional CMV promoter (2xCMV), and the other with the eCMV cassette (2xeCMV). Interestingly, the 2xCMV construct did not result in increased *lc* or *hc* mRNA levels compared to the bidirectional construct containing two identical eCMV promoters or constructs containing two different promoters. The BiDi combination of two mirrored eCMV promoter cassettes (2xeCMV) yielded in a 7-fold upregulation of *lc* mRNA and a modest upregulation in *hc* levels.

**Figure 4:**
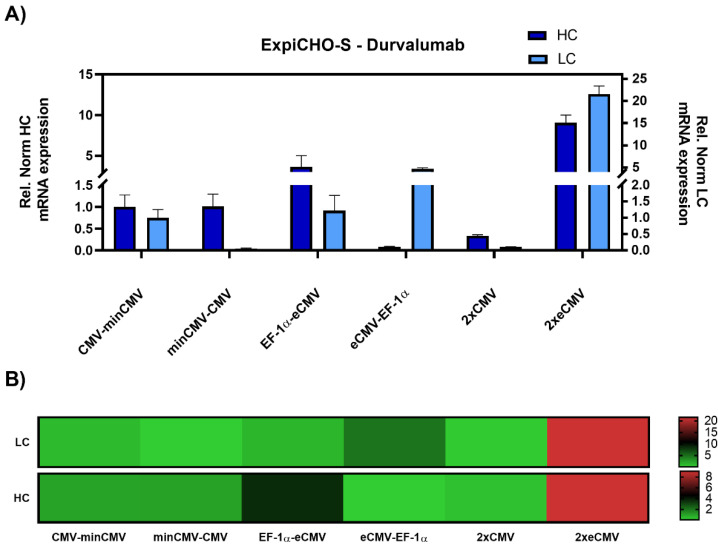
Gene expression analysis of heavy and light chain genes after transient transfection of Durvalumab in ExpiCHO-S cells. (**A**) Bar chart representing heavy (dark blue) and light (light blue) chain mRNA expression in the different constructs. Values are relative to the CMV-minCMV construct and normalised to housekeeping genes GAPDH and RPLP0. Error bars represent the standard error of the mean of technical triplicates. (**B**) Heat map representation of gene expression analysis. The relative normalised gene expression for light and heavy chain mRNA is shown on the right with their respective scales.

As promoter strength may vary depending on the cell line, particularly for the CMV promoter [33], ExpiCHO-S cells were also investigated. Gene expression analysis resulted in similar results as in Expi293-F cells, with the 2xeCMV complex showing the strongest upregulation in both *hc* and *lc* mRNA levels compared to the other promoter combination, namely a modest 10-fold upregulation of *hc*, and a 20-fold upregulation of *lc* mRNA (Figure 4).

### 3.3. Protein Yield Determination via BLI

**Figure 5:**
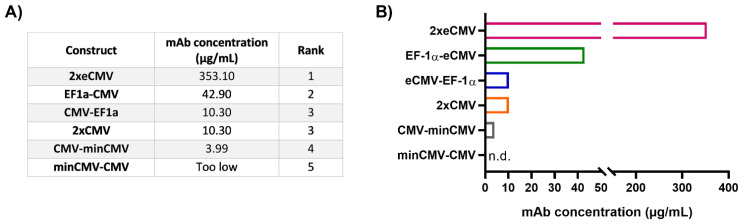
Protein quantification of Durvalumab in cell culture supernatants from transfected Expi293-F cells. (**A**) Table showing the mAb concentrations from 24-well transient transfections, listed according to their rank. The ranks 1–5 were set based on the mAb concentration of the different BiDi combinations. (**B**) Bar chart representation of BiDi mAb concentrations.

As mRNA transcript levels do not indicate successful secretion of fully functional recombinant antibodies, protein quantification studies were performed. Expi293-F cells were transiently transfected and the amount of secreted therapeutic antibody Durvalumab [27] was quantified by biolayer interferometry (BLI) using sterile-filtered cell culture supernatants six days post-transfection. The mAb concentrations for the different constructs were interpolated from a standard curve generated using an in-house produced mAb. In line with the gene expression analysis, protein quantification resulted in a clear ranking of the different promoter combinations (Figure 5).

From the bidirectional combinations tested with transient transfections, the 2xeCMV BiDi construct showed the highest mAb concentration with 353 µg/mL (Figure 5). Interestingly, mRNA expression was higher for EF-1α-eCMV compared to 2xeCMV for both *hc* and *lc*, but antibody yield was significantly reduced in Expi293-F cells (Figure 3). This may likely be due to the fact that EF-1α-eCMV displayed similar *hc* and *lc* mRNA expression patterns, while 2xeCMV revealed a higher accumulation of the *lc* compared to the *hc* mRNA. It is well known that the expression of excess light chain over heavy chain is often beneficial for antibody production [34,35,36,37]. These findings corroborate that 2xeCMV resulted in the most promising bidirectional promoter combination, particularly in view of the fact that the usage of this promoter combination resulted, both in Expi293-F and ExpiCHO-S cell lines, in significantly enhanced mRNA synthesis with an excess of *lc* over *hc*.

### 3.4. Correlation of mRNA and Protein Levels

**Figure 6:**
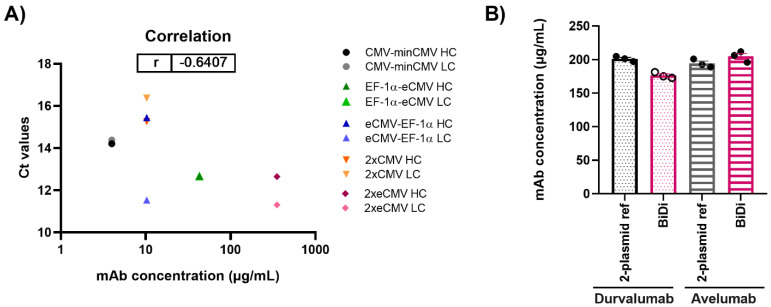
(**A**) Correlation of mAb concentration and Ct values for both heavy and light chain expression in Expi293-F for production of Durvalumab. (**B**) Quantification of antibody concentration for the production of Durvalumab and Avelumab using either a 2-plasmid reference or the BiDi 2xeCMV construct. Error bars represent the standard error of the mean of biological triplicates, while the symbols represent the individual measurements.

After performing both mRNA and protein studies, the correlation of the cycle threshold (Ct) values from RT-qPCR and the mAb concentrations from protein quantification in Expi293-F was determined (Figure 6A). Overall, a Pearson’s coefficient of −0.6407 was calculated, indicating, as expected, a negative correlation between Ct values and mAb concentration. Looking at the respective Ct values of either the *hc* or *lc* for 2xeCMV, one can observe they have a low Ct value and resulted in the largest mAb yield. While some claim that abundance of *hc* may hinder productivity, it appears that the excess of *lc* allowing for correct mAb folding and assembly is sufficient for higher mAb yields. On the contrary, the Ct values of eCMV-EF-1α *lc* is similar to that of 2xeCMV, with the only difference being in the *hc* expression, ultimately resulting in much lower yields. Thus, this further shows the importance of a fine-tuned *hc* and *lc* expression, substantiating the potential of 2xeCMV for a BiDi antibody production system.

### 3.5. Antibody—And Light Chain-Independence

To ensure the promoter combination used for our BiDi technology was not antibody- or light chain isotype-dependent, the FDA-approved anti-PD-L1 antibody Avelumab of the λ isotype was also tested [38]. As 70% of approved antibodies belong to the κ type [39,40], only the most promising 2xeCMV construct was used to validate the established system for a λ-based mAb, compared to the conventional co-transfection approach. For co-transfection, Expi293-F cells were transfected using a 1:1 ratio of HC and LC DNA, with each plasmid carrying the same promoter and enhancer cassette as the bidirectional vector, namely eCMV. As can be appreciated from Figure 6B, there was no significant variation in antibody yield between transfections with BiDi and the two-plasmid reference for either Durvalumab or Avelumab. Similarly, no variation was observed in ExpiCHO-S production (data not shown). Kinetics determination of both antibodies produced with either the two-plasmid reference or our established 2xeCMV BiDi plasmid bound to its target PD-L1 with comparable affinities (Table 1, Figures S1 and S2). Additionally, SDS-PAGE analysis under reducing conditions resulted in the expected heavy and light chains bands (Figure S3).

Thus, this indicated the established system is compatible with different binders and can be employed for both κ- and λ-light chain isotypes.

## 4. Discussion

This work describes the generation of a bidirectional vector construct for recombinant antibody expression using two eCMV promoter cassettes, controlling the expression of *lc* and *hc* individually in each direction. By performing a thorough analysis of different bidirectional promoter combinations with varying lengths and strengths, the 2xeCMV combination showed the most promising *hc* and *lc* mRNA synthesis in two regularly used mammalian cell lines, and, more importantly, the highest yields after protein quantification comparable to those using the conventional two-plasmid system. Other BiDi constructs showed potentially promising gene expression profiles, such as EF-1α-eCMV combination, with high relative mRNA levels of both *hc* and *lc* mRNA. Nonetheless, having higher levels of *hc* mRNA in the cells appear to curb productivity of fully folded IgG formation, resulting in drastically decreased antibody yields compared to both the two-plasmid reference and the 2xeCMV BiDi vector.

While using a bidirectional approach takes away some flexibility in terms of being able to alter the *hc:lc* ratios during co-transfection, the overexpression of both genes and, especially the excess *lc* expression, results in sufficient material for antibody hit screening. For convenience, we established a one-step cloning procedure for simultaneous plasmid incorporation of the heavy and light chain encoding segments obviating the need for generating two separate plasmids. Not only does this approach lower plasmid preparation efforts, but it also increases handling for transfection of numerous mAbs during screening and characterization as only a single plasmid is required. The use of two FDA-approved antibodies with either a κ- or λ-light chain shows there is no antibody- or light chain-dependence using this system, indicating that it can be implemented ubiquitously. Further options remain to increase the yields of IgG molecules, such as optimization of the stuffer region between the two eCMV promoter cassettes, potentially reducing any steric hinderance and increasing transcription efficiency [22,41].

In conclusion, this work displays the benefits of using a one-plasmid bidirectional system with 2xeCMV promoters for fully folded IgGs within drug discovery. In terms of practicality, handling of a single plasmid for antibody production may be superior to the conventional way. Moreover, yields of fully folded IgGs are comparable between the two systems. Future directions for this technology go beyond recombinant production of classical antibody formats, as reduction of the number of plasmid constructs could also be considered feasible for the expression of bispecific antibodies and other antibody formats in the frame of antibody drug discovery.

## Figures and Tables

**Table 1:** Affinity determination of Avelumab and Durvalumab using either co-transfection or 2xeCMV BiDi plasmid.

	Antibody	K_D_ [pM]	
		2-Plasmid Ref.	BiDi
Expi293-F	Durvalumab	594	364
	Avelumab	205	195
ExpiCHO-S	Durvalumab	562	522
	Avelumab	148	267

## Data Availability

The data presented in this study are available within this article and its Supplementary Materials.

## Supplementary Materials

The following are available online at https://www.mdpi.com/article/10.3390/antib10020018/s1, Table S1: Primers used for cloning of bidirectional constructs, Table S2: RT-qPCR primers for HC and LC constant regions, Figure S1: Affinity determination by BLI of antibodies produced in Expi293-F, Figure S2: Affinity determination by BLI of antibodies produced in ExpiCHO-S, Figure S3: SDS-PAGE analysis of purified antibodies, Sequence S1: DNA sequence of the designed Durvalumab-2xeCMV insert.
