# Supplementary material for: Recombinant Antibody Production Using a Dual-Promoter Single Plasmid System
Source: Antibodies (Basel). 2021 May 13;10(2):18. doi: 10.3390/antib10020018 (PMC8161450; doi:10.3390/antib10020018)
Supplement: Supplementary file 1 [file antibodies-10-00018-s001.zip › antibodies-1176496-SI/antibodies-1176496-SI.pdf]

Supplementary Information

Recombinant antibody production using a dual-promoter single plasmid system

Stefania C. Carrara+, David Fiebig+, Jan P. Bogen+, Julius Grzeschik, Björn Hock, and Harald Kolmar

Table S1: Primers used for cloning of bidirectional constructs

AvelumabVH_SapI_for	AAAAAGCTCTTCAAGTGAAGTTCAGCTGTTGG
AvelumabVH_SapI_rev	TTTTTTGCTCTTCTGGCAGAGGAGACAGTAACAAGAG
AvelumabVL_SapI_for	AAAAAGCTCTTCAAGTCAATCCGCCTTGACTC
AvelumabVL_SapI_rev	TTTTTTTTGCTCTTCACCCGAGAACTGTGACCTTTG
DurvalumabVH_SapI_for	AAAAAGCTCTTCAAGTGAGGTTCAACTTGTTGAAAGCGG
DurvalumabVH_SapI_rev	TTTTTTGCTCTTCTGGCGCTTGAGACTGTAACGAGGG
DurvalumabVL_SapI_for	AAAAAGCTCTTCAAGTGAAATAGTGCTTACCCAAAGTCC
DurvalumabVL_SapI_rev	TTTTTTGCTCTTCATCGTTTAATTTCGACCTTAGTAC
MD-Leader-VH_BbsI_for	ATATAGAAGACATCGCTTGCCACCATGAC
CH1_MD_Esp3I_rev	ATATATCGTCTCGGTATGGGTCTTGTCGCAGCTCTTGG
MD-Leader-VL_BbsI_for	ATATAGAAGACCGTCGCAGCCACCATGAC
Lam-CL_Esp3I_rev	ATATACGTCTCGAGATCTATTAGCTGCACTCGGTGGGGGCCACGGTTTT CTCCACGGTGCTGCCCTC
Kap-CL_Esp3I_rev	ATATACGTCTCGAGATCTATTAACACTCTCCCCTGTTGAAGCTC
eCMV-HC_BbsI_rev	GCGCGGAAGACATAGCGCGCTAGAGATCCGTTTAAACTTGG
eCMV-LC_BbsI_rev	GCGCGGAAGACATGCGACGCTAGAGATCCGTTTAAACTTGG
eCMV_LC-Stuffer-Con_BbsI_for	GCGCGGAAGACATGATGGTACATTTATATTGGCTCATGTCCAATATGAC CGC
eCMV_HC-Stuffer-Con_BbsI_for	GCGCGGAAGACATGGCTGTACATTTATATTGGCTCATGTCCAATATGAC CGC
CMV-HC_BbsI_rev	GCGCGGAAGACATAGCGGATCTGACGGTTCACTAAACCAG
CMV-HC-Stuffer-Con_BbsI_for	GCGCGGAAGACATGGCTCCGCGTTACATAACTTACGGTAAATG
CMV-LC-BbsI_rev	GCGCGGAAGACATGCGAGATCTGACGGTTCACTAAACCAGC
CMV-LC-Stuffer-Con_BbsI_for	GCGCGGAAGACATGATGCCGCGTTACATAACTTACGGTAAATG
EF1a-LC-Stuffer-Con_BbsI_for	GCGCGGAAGACATGATGGGC
EF1a-LC_BbsI_rev	ATATAGAAGACATGCGAAGCCTGCTTTTTTGTACAAACTTGTCAC
HC-minCMV-CMV-LC_BbsI_rev	GCGCGGAAGACTAAGCGTCTGACGGTTCACTAAACGAGCTCTGC
HC-minCMV-CMV-LC_BbsI_for	GCGCGGAAGACATGCGAGATCTGACGGTTCACTAAACCAGC

Table S2: RT-qPCR primers for HC and LC constant regions

HC constant_fwd	TTCCAGAACCAGTCACCGTT
HC constant_rev	CCAAAGAAGAAGAGGGAACA
LC kappa constant_fwd	TGTAGGTGCTGTCCTTGCTG
LC kappa constant_rev	CTGTTGTGTGCCTGCTGAAT
LC lambda constant_fwd	GTAGCTCCTGTGGCTTTTC
LC lambda constant_rev	TGATCAGCGACTTCTACCC

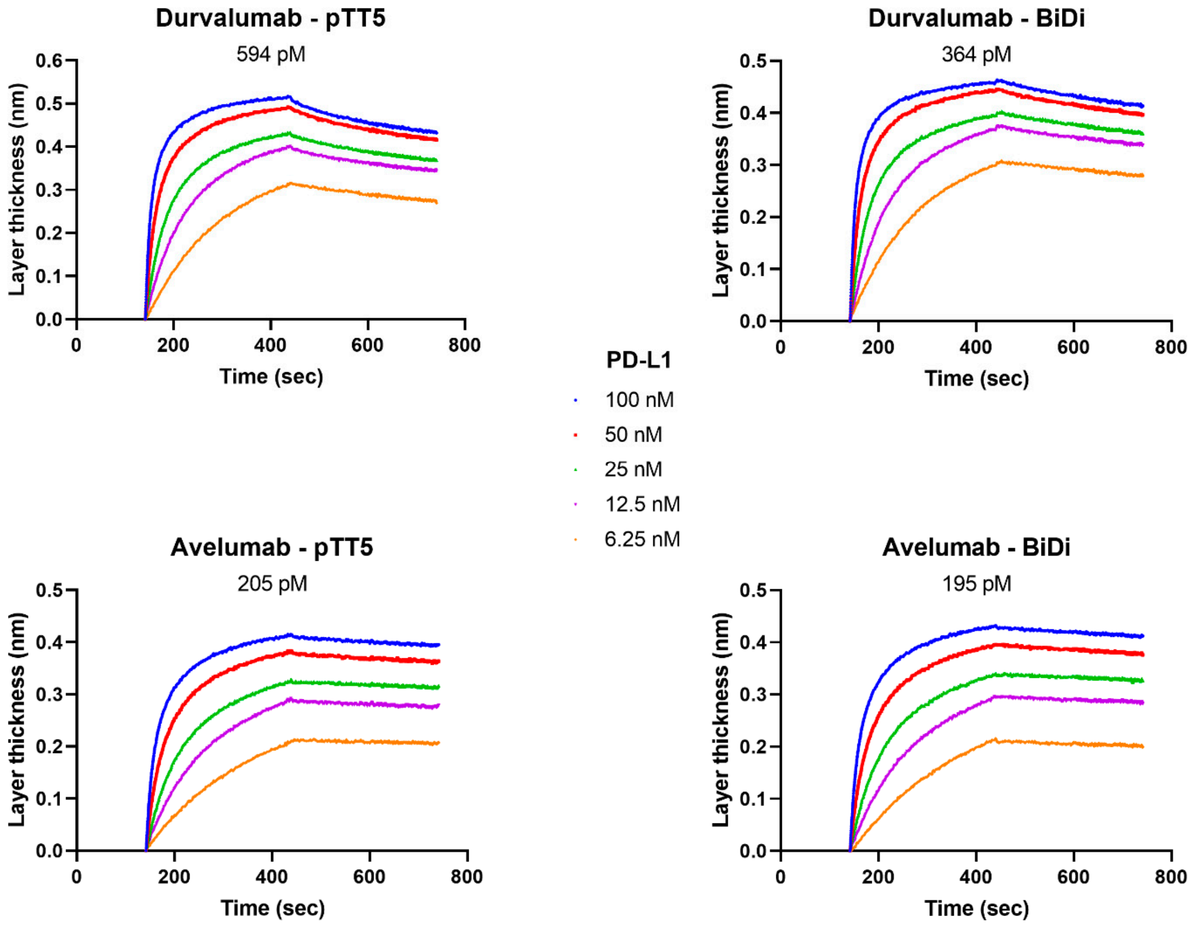

Figure S1: Affinity determination by BLI of antibodies produced in Expi293-F. Binding of Durvalumab and Avelumab produced in Expi293-F cells to PD-L1. Antibodies were either produced by co-transfection (denoted as pTT5) or transfection using 2xeCMV BiDi construct (denoted as BiDi). The curves represent binding of 10 µg/mL antibody to different concentrations of PD-L1.

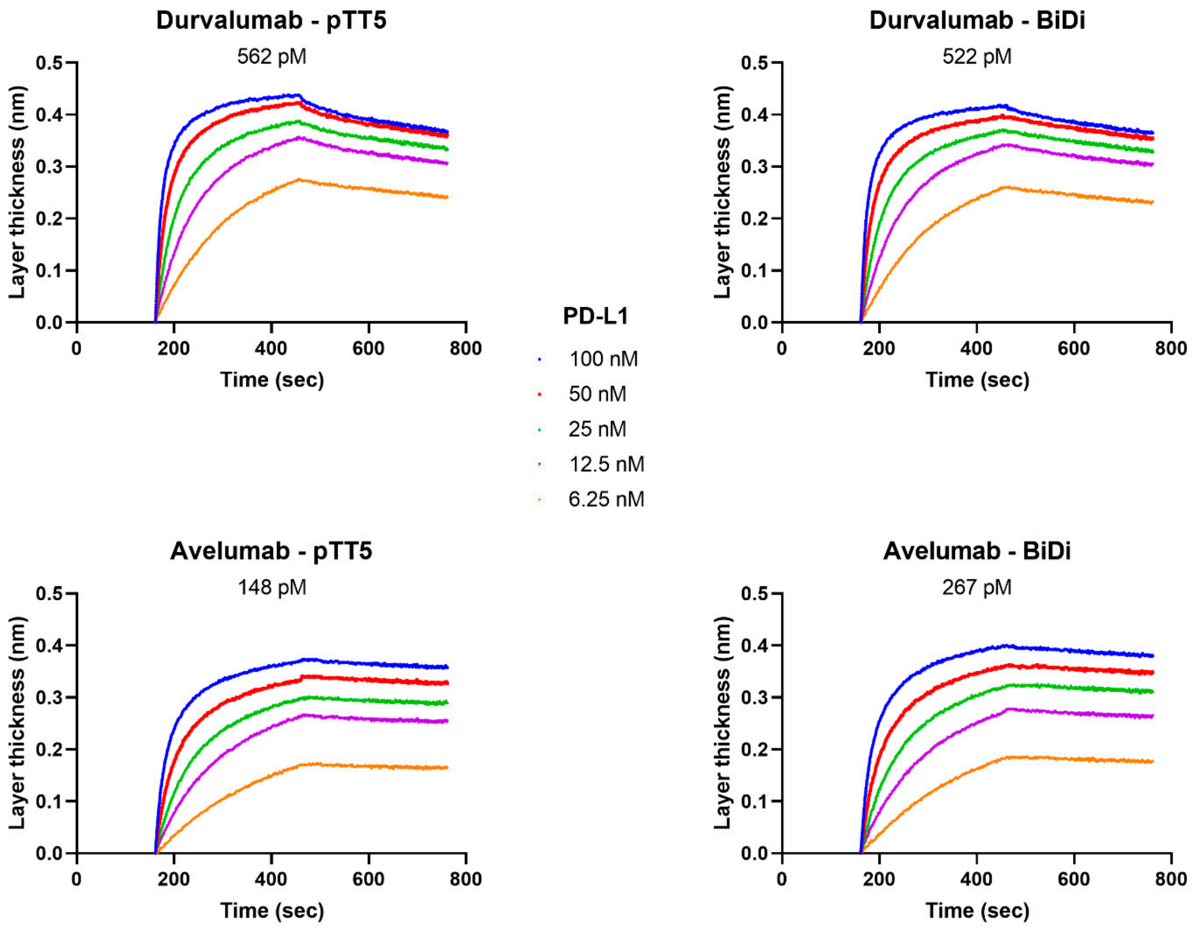

Figure S2: Affinity determination by BLI of antibodies produced in ExpiCHO-S. Binding of Durvalumab and Avelumab produced in ExpiCHO-s cells to PD-L1. Antibodies were either produced by co-transfection (denoted as pTT5) or transfection using 2xeCMV BiDi construct (denoted as BiDi). The curves represent binding of 10 µg/mL antibody to different concentrations of PD-L1.

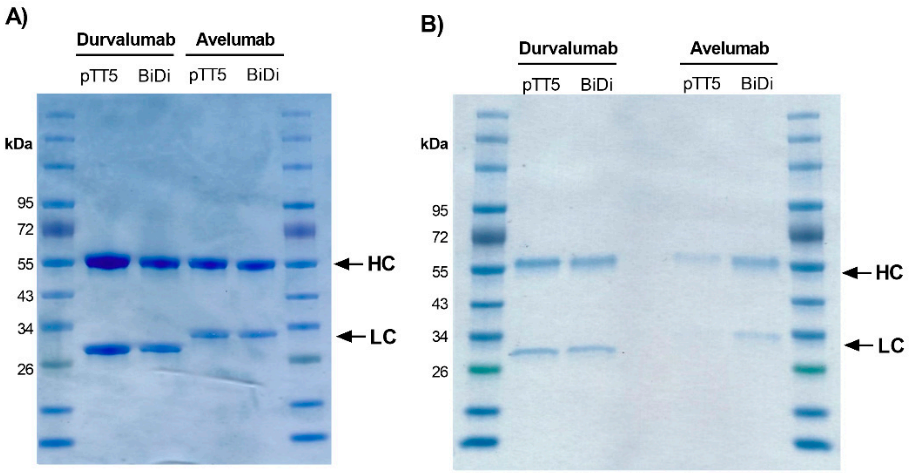

Figure S3: SDS-PAGE analysis of purified antibodies. Heavy and light chain bands at their expected molecular weights. A) represents purified antibodies from an Expi293-F production, while B) represents those produced in ExpiCHO-S cells. After purification, 3 µg or 0.5 µg antibodies were loaded onto an SDS-PAGE gel after Expi293-F and ExpiCHO-S production and purification, respectively.

Sequence S1: DNA sequence of the designed Durvalumab-2xeCMV insert. The colour codes represent the following regions: SV40 polyA signal (grey), CL Kappa (cyan), VL Durvalumab (magenta), Signal peptide (brown), Enh. MLP (dark green), TLP (red), CMV promoter (yellow), CMV Enhancer (green), Stuffer (blue), VH Durvalumab (dark blue), CH1-CH2-CH3 (black), Stop codon (dark yellow).

CGGCAGTGAAAAAAATGCTTTATTTGTGAAATTTGTGATGCTATTGCTTTATTTGTAACCATTATAAGCTGCAATAAAC
AAGTTAACAACAACAATTGCATTCATTTTATGTTTCAGGTTCAGGGGGAGGTGTGGGAGGTTTTTTAAAGCAAGTAAA
ACCTCTACAAATGTGGTATGGCTGATTATGAGCTAGAGATCTATTAACACTCTCCCCTGTTGAAGCTCTTTGTGACGGG
CGAGCTCAGGCCCTGATGGGTGACTTCGCAGGCGTAGACTTTGTGTTTCTCGTAGTCTGCTTTGCTCAGCGTCAGGGT
GCTGCTGAGGCTGTAGGTGCTGTCCTTGCTGTCCTGCTCTGTGACACTCTCCTGGGAGTTACCCGATTGGAGGGCGTT
ATCCACCTTCCACTGTACTTTGGCCTCTCTGGGATAGAAGTTATTCAGCAGGCACACAACAGAGGCAGTTCCAGATTTC
AACTGCTCATCAGATGGCGGGAAGATGAACACAGATGGTGCAGCCACAGTTCGTTTAATTTCGACCTTAGTACCTTGG
CCGAATGTCCAAGGGAGACTACCATATTGTTGACAGTAGTAGACTGCAAAATCTTCCGGTTCCAGCCGGGAAATTGTA
AGTGTAAAATCTGTGCCGGAACCGCTTCCTGAAAACCTATCAGGTATGCCAGTAGCTCTAGAGGATGCGTCATAGATC
AACAGACGCGGTGCTTGCCCAGGCTTTTGTTGATACCATGCAAGATATGAACTGGATACCCTTTGTGAAGCTCTGCAA
GACAGAGTTGCTCTTTCACCCGGGGAGAGACTCAGGGTGCCGGGACTTTGGGTAAGCACTATTTCAGCCAAGGCAAT
ATTAGTTAATCCCAACAATATTGTGAACAGGTAGGTAAAATGAGCGAAAGATAATGTCATGGTGGCTGCGACGCTAG
AGATCCGTTTAAACTTGGACCTGGGAGTGGACACCTGTGGAGAGAAAGGCAAAGTGGATGTCATTGTCACTCAAGTG
TATGGCCAGATCGGGCCAGGTGAATATCAAATCCTCCTCGTTTTTGGAAACTGACAATCTTAGCGCAGAAGTAATGCC
CGCTTTTGAGAGGGAGTACTCACCCCAACAGCTGGATCTCAAGCCTGCCACACCTCACCTCGACCATCCGCCGTCTAAA
GACCGCCTACTTTAATTACATCATCAGCAGCACCTCCGCCAGAAACAACCCCGACCGCCACCCGCTGCCGCCCGCCACG
GTGCTCAGCCTACCTTGCGACTGTGACTGGTTAGACGCCTTTCTCGAGAGGTTTTCCGATCCGGTCGATGCGGACTCG
CTCAGGTCCCTCGGTGGCGGAGTACCGTTCGGAGGCCGACGGGTTTCCGATCCAAGAGTACTGGAAAGACCGCGAA
GAGTTTGTCCTCAACCGCGAGCCCAACAGCTGGCCCTCGCAGACAGCGATGCGGAAGAGAGTGAGGATCTGACGGTT
CACTAAACGAGCTCTGCTTATATAGACCTCCCACCGTACACGCCTACCGCCCATTTGCGTCAACGGGGCGGGGTTATTA

CGACATTTTGGAAAGTCCCGTTGATTTTGGTGCCAAAACAAACTCCCATTGACGTCAATGGGGTGGAGACTTGGAAAT
CCCCGTGAGTCAAACCGCTATCCACGCCCATTGGTGTACTGCCAAAACCGCATCACCATGGTAATAGCGATGACTAAT
ACGTAGATGTACTGCCAAGTAGGAAAGTCCCGTAAGGTCATGTACTGGGCATAATGCCAGGCGGGCCATTTACCGTC
ATTGACGTCAATAGGGGGCGGACTTGGCATATGATACACTTGATGTACTGCCAAGTGGGCAGTTTACCGTAAATACTC
CACCCATTGACGTCAATGGAAAGTCCCTATTGGCGTTACTATGGGAACATACGTCATTATTGACGTCAATGGGCGGGG
GTCGTTGGGCGGTCAGCCAGGCGGGCCATTTACCGTAAGTTATGTAACGCGGAACTCCATATATGGGCTATGAACTA
ATGACCCCGTAATTGATTACTATTAATAACTAGTCAATAATCAATGTCAACATGGCGGTCATATTGGACATGAGCCAAT
ATAAATGTACCATCTAAAGTATATATGAGTAAACTTGGTCTGACAGTTACCAATGCTTAATCAGTGAGGCACCTATCTC
AGCGATCTGTCTATTTCGTTCATCCATAGTTGCCTGACTCCCCGTCGTGTAGATAACTACGATACGGGAGGGCTTACCA
TCTGGCCCCAGTGCTGCAATGATACCGCGAGATCCACGCTCACCGGCTGTACATTTATATTGGCTCATGTCCAATATGA
CCGCCATGTTGACATTGATTATTGACTAGTTATTAATAGTAATCAATTACGGGGTCATTAGTTCATAGCCCATATATGG
AGTTCCGCGTTACATAACTTACGGTAAATGGCCCGCCTGGCTGACCGCCCAACGACCCCCGCCCATTGACGTCAATAA
TGACGTATGTTCCCATAGTAACGCCAATAGGGACTTTCCATTGACGTCAATGGGTGGAGTATTTACGGTAAACTGCCC
ACTTGGCAGTACATCAAGTGTATCATATGCCAAGTCCGCCCCCTATTGACGTCAATGACGGTAAATGGCCCGCCTGGC
ATTATGCCCAGTACATGACCTTACGGGACTTTCCTACTTGGCAGTACATCTACGTATTAGTCATCGCTATTACCATGGTG
ATGCGGTTTTGGCAGTACACCAATGGGCGTGGATAGCGGTTTGACTCACGGGGATTTCCAAGTCTCCACCCCATTGAC
GTCAATGGGAGTTTGTTTTGGCACCAAAATCAACGGGACTTTCCAAAATGTCGTAATAACCCCGCCCCGTTGACGCAA
ATGGGCGGTAGGCGTGTACGGTGGGAGGTCTATATAAGCAGAGCTCGTTTAGTGAACCGTCAGATCCTCACTCTCTTC
CGCATCGCTGTCTGCGAGGGCCAGCTGTTGGGCTCGCGGTTGAGGACAAACTCTTCGCGGTCTTTCCAGTACTCTTGG
ATCGGAAACCCGTCGGCCTCCGAACGGTACTCCGCCACCGAGGGACCTGAGCGAGTCCGCATCGACCGGATCGGAAA
ACCTCTCGAGAAAGGCGTCTAACCAGTCACAGTCGCAAGGTAGGCTGAGCACCGTGGCGGGCGGCAGCGGGTGGCG
GTCGGGGTTGTTTCTGGCGGAGGTGCTGCTGATGATGTAATTAAAGTAGGCGGTCTTTAGACGGCGGATGGTCGAGG
TGAGGTGTGGCAGGCTTGAGATCCAGCTGTTGGGGTGAGTACTCCCTCTCAAAAGCGGGCATTACTTCTGCGCTAAG
ATTGTCAGTTTCCAAAAACGAGGAGGATTTGATATTCACCTGGCCCGATCTGGCCATACACTTGAGTGACAATGACAT
CCACTTTGCCTTTCTCTCCACAGGTGTCCACTCCCAGGTCCAAGTTTAAACGGATCTCTAGCGCGCTTGCCACCATGACA
TTATCTTTCGCTCATTTTACCTACCTGTTCACAATATTGTTGGGATTAACTAATATTGCCTTGGCAGAGGTTCAACTTGTT
GAAAGCGGCGGTGGTCTGGTCCAACCTGGTGGTTCCTTGCGCCTGAGTTGCGCCGCTAGTGGCTTCACTTTTAGTCGA
TATTGGATGTCCTGGGTACGCCAAGCACCTGGCAAGGGCTTGGAATGGGTGGCTAATATTAAGCAAGATGGATCAGA
GAAATACTATGTCGACAGCGTAAAAGGGCGGTTTACTATTTCTAGGGACAACGCAAAGAACTCCCTGTACCTTCAAAT
GAACTCTCTTCGCGCTGAGGACACTGCTGTGTACTACTGCGCCAGGGAGGGAGGTTGGTTCGGAGAACTGGCATTCG
ATTATTGGGGCCAGGGCACCCTCGTTACAGTCTCAAGCGCCTCTACTAAAGGTCCATCTGTTTTTCCATTGGCTCCATCT
TCTAAATCTACATCTGGTGGTACTGCTGCTTTGGGTTGTTTGGTTAAGGATTATTTCCAGAACCAGTCACCGTTTCTTG
GAATTCTGGTGCTTTGACTTCTGGTGTTCATACTTTTCCAGCCGTATTGCAATCTTCTGGCTTGTATTCTTTGTCCTCTGT
TGTTACTGTTCCCTCTTCTTCTTTGGGTACTCAAACTTACATCTGCAACGTTAACCATAAGCCATCTAACACCAAGGTTG
ATAAGAGAGTCGAACCTAAGTCTTGTGATAAGACTCATACCTGTCCCCCTTGTCCTGCCCCTGAAGCCGCCGGCGGAC
CTTCCGTGTTCCTGTTCCCCCCAAAGCCCAAGGATACCCTGATGATCAGCCGGACCCCCGAAGTGACCTGCGTGGTGG
TGGATGTGTCCCACGAGGACCCTGAAGTGAAGTTCAATTGGTACGTGGACGGCGTGGAAGTGCACAACGCCAAGACC
AAGCCCAGAGAGGAACAGTACAACAGCACCTACCGGGTGGTGTCCGTGCTGACAGTGCTGCATCAGGACTGGCTGAA
CGGCAAAGAGTACAAGTGCAAGGTGTCCAACAAGGCCCTGCCTGCCCCCATCGAGAAAACCATCAGCAAGGCCAAGG
GCCAGCCCCGCGAACCCCAGGTGTACACACTGCCTCCCAGCAGGGACGAGCTGACCAAGAACCAGGTGTCCCTGACC
TGTCTCGTGAAAGGCTTCTACCCCTCCGATATCGCCGTGGAATGGGAGAGCAATGGCCAGCCCGAGAACAACTACAA
GACCACCCCCCCTGTGCTGGACAGCGACGGCTCATTCTTCCTGTACAGCAAGCTGACCGTGGACAAGTCCCGGTGGCA
GCAGGGCAACGTGTTCAGCTGCAGCGTGATGCACGAGGCCCTGCACAACCACTACACCCAGAAGTCCCTGAGCCTGA
GCCCCGGCAAATAATGATCCAGAGGATCATAATCAGCCATACCACATTTGTAGAGGTTTTACTTGCTTTAAAAAACCTC
CCACACCTCCCCCTGAACCTGAAACATAAAATGAATGCAATTGTTGTTGTTAACTTGTTTATTGCAGCTTATAATGGTTA
CAAATAAAGCAATAGCATCACAAATTTCACAAATAAAGCATTTTTTTCACTGCCCCGTGCGGTGGG
